# Molecular Subtyping of Alzheimer's Disease with Mixed Pathology from a Single Nucleus/Cell Transcriptomics Atlas

**DOI:** 10.1002/alz70855_103590

**Published:** 2025-12-23

**Authors:** Negin Rahimzadeh, Samuel Morabito, Zechuan Shi, Vivek Swarup

**Affiliations:** ^1^ Center for Complex Biological Systems (CCBS), UC Irvine, Irvine, CA, USA; ^2^ Mathematical, Computational and Systems Biology (MCSB) Program, UC Irvine, Irvine, CA, USA; ^3^ University of California, Irvine, Irvine, CA, USA; ^4^ Institute for Memory Impairments and Neurological Disorders, Irvine, CA, USA; ^5^ University of California, Los Angeles School of Medicine, Los Angeles, CA, USA; ^6^ Institute for Memory Impairments and Neurological Disorders (MIND), Irvine, CA, USA

## Abstract

**Background:**

Alzheimer's disease (AD) is currently an incurable and heterogeneous neurodegenerative disorder. Traditionally, AD is diagnosed through clinical evaluation alongside pathological hallmarks, including amyloid‐beta plaques and tau tangles. However, discrepancies exist wherein individuals exhibit AD pathology without clinical symptoms or are diagnosed clinically without classical pathological features. Recent studies have also explored heterogeneity across gender and race, further emphasizing the disease's complexity and the need for subtype‐specific therapeutic approaches.

**Method:**

To address AD heterogeneity, we integrated 12 datasets to construct a mixed‐pathology AD atlas (*n* = 876), categorizing samples into three groups: Control, Mild Cognitive Impairment (MCI), and mixed‐pathology Alzheimer's. Differential expression analysis, hierarchical density‐weighted co‐expression network analysis, CellChat, and SCENIC algorithms were employed to characterize inter‐group differences. An autoencoder approach was used to identify sample clusters within the AD group, leveraging latent low‐dimensional representations of gene expression patterns. Gradient‐based analysis ranked genes by their contributions to embeddings, and the top 400 genes per cluster were analyzed using EnrichR to identify enriched pathways.

**Result:**

The K‐means algorithm was applied to the embeddings with optimal k chosen as 5, determined by the Elbow Method, to assign cluster labels to the data points. This approach generated five robust AD mixed pathology subtypes, characterized by GO terms: AD0 Pathogen Defense and Immune Regulation, AD1 NF‐kappaB and Cytokine Signaling, AD2 CNS Development and Axon Guidance, AD3 Lipid Metabolism and Reactive Oxygen Regulation, and AD4 Neurodevelopment and Cellular Adhesion. All AD subgroups exhibited distinct patterns across ApoE genotype, tissue distribution, and Braak stage. The AD3 and AD4 groups showed the highest representation in the E3/E3 and E3/E4 genotypes, a strong association with prefrontal cortex and middle temporal gyrus tissues, and dominance in Braak stages 5 and 6.

**Conclusion:**

Constructing an AD atlas at a single‐cell/nucleus resolution enabled us to identify changes specific to AD compared to MCI and Control samples. Furthermore, we defined novel AD subtypes using a simple autoencoder‐based classification approach. These findings provide a foundation for subtype‐specific research and therapeutic development in AD.

